# The quality and reliability of short videos about Marfan syndrome on Bilibili and Douyin: a cross-sectional study

**DOI:** 10.7717/peerj.21843

**Published:** 2026-10-25

**Authors:** Tao Sun, Jing Hu

**Affiliations:** Department of Obstetrics, Anji Maternal and Child Health Hospital, Anji, Zhejiang, China

**Keywords:** Cross-sectional, Marfan syndrome, Social media, Douyin, Bilibili, Medical education

## Abstract

**Background:**

Marfan syndrome (MS) is a rare, life-threatening connective tissue disorder. Short video platforms are now key health-information sources, yet the quality and reliability of MS-related content remain unclear.

**Methods:**

In a cross-sectional study, Marfan syndrome videos were retrieved from Bilibili and Douyin on 25 April 2026 using the Chinese keyword “马凡综合征,” from new accounts under anonymous browsing. After screening, data on video characteristics, uploader type, and publicly displayed likes, comments, shares, and collections were extracted. Two reviewers independently scored all eligible videos with Global Quality Scale (GQS), modified Decision-making Information Support Criteria for Evaluating the Reliability of Non-randomised Studies (mDISCERN), Journal of the American Medical Association (JAMA) criteria, and Content Completeness Score (CCS). Descriptive statistics, non-parametric tests, Spearman correlations, and weighted Cohen’s kappa were used.

**Results:**

185 videos (103 from Douyin and 82 from Bilibili) were analyzed. The median video duration was significantly longer on Bilibili than on Douyin (126.00 (IQR: 59.00–377.00) *vs.* 64.00 (IQR: 45.50–103.50) seconds; *P* < 0.001). Douyin videos had been available for a significantly shorter time since publication (median: 275.00 *vs.* 747.50 days; *P* < 0.001), yet their per-day interaction counts remained significantly higher after this adjustment (all *P* < 0.001). Douyin videos demonstrated significantly higher absolute interaction counts, including likes (median: 2,214.00 *vs.* 48.00; *P* < 0.001), comments (240.00 *vs.* 0.00; *P* < 0.001), shares (606.00 *vs.* 5.50; *P* < 0.001), and collections (475.00 *vs.* 24.00; *P* < 0.001). In terms of quality, Douyin videos had higher GQS scores than Bilibili (median: 4.00 *vs.* 3.00; *P* < 0.001), whereas Bilibili videos showed higher mDISCERN scores (median: 3.00 *vs.* 2.00; *P* < 0.001). No significant differences were observed in JAMA scores (*P* = 0.245) or CCS (*P* = 0.274). Healthcare professionals accounted for the majority of Douyin content (78.6%), while institutional sources predominated on Bilibili (53.7%) (*P* < 0.001). Videos produced by professionals and institutions achieved significantly higher quality and reliability scores compared to individual uploaders. Spearman correlation analysis revealed strong correlations among engagement metrics (*r* = 0.84–0.94), but weak or negligible correlations between absolute interaction counts and quality scores (*r* = −0.16 to 0.17).

**Conclusion:**

Marfan syndrome-related videos on Bilibili and Douyin varied considerably in educational quality and reliability; those by healthcare professionals were generally higher quality. Within this sample, observed interaction counts showed weak or negligible associations with quality measures.

## Introduction

Marfan syndrome (MS) is an autosomal dominant connective tissue disorder caused by mutations in the *FBN1* gene (Milewicz et al., 2021; Muino-Mosquera et al., 2024). It affects about 1 in 5,000 individuals worldwide. This disorder predominantly involves cardiovascular, skeletal, and ocular systems, with progressive aortic root dilation and acute aortic dissection representing the most life-threatening complications (Milewicz et al., 2021; Muino-Mosquera et al., 2024). Due to marked clinical variability and overlapping features with other disorders, diagnostic delays are common, and many patients remain undiagnosed until a catastrophic cardiovascular event occurs (Muino-Mosquera et al., 2024). Lifelong multidisciplinary management, including regular imaging surveillance, anti-hypertensive therapy, and elective prophylactic aortic surgery, is critical to survive (Milewicz et al., 2021; Muino-Mosquera et al., 2024). Given this complexity, health literacy and access to accurate, comprehensible information are essential for patient adherence to monitoring and lifestyle modifications.

Nowadays, the Internet has become a primary source of health information globally, with YouTube exceeding 2.5 billion monthly active users and TikTok surpassing 1 billion (Cakici & Koseoglu, 2025; Zhao & Liu, 2026). Video-sharing content on these platforms increasingly serves as a channel for disseminating medical knowledge, including content on rare diseases (Buschulte et al., 2024; Zhu et al., 2026). In China, social media platforms are also popular among Internet users (Liu et al., 2026). Bilibili is one of the most popular Chinese video-sharing platforms known for its interactive features and longer-duration and professional content, attracting a large user base (Liu et al., 2026). Douyin, the Chinese version of TikTok, has attracted more than 200 million active users daily (Gong et al., 2025). However, unlike peer-reviewed medical studies, social media platforms lack rigorous content review mechanisms. This may lead to the dissemination of low-quality or misleading health videos (Gulmez & Ciftci, 2026; Zhao & Liu, 2026). Previously, studies reported that health misinformation on social media can adversely affect patient understanding and health-related decision-making (Akca Sumengen et al., 2026; Liu et al., 2026).

Despite the increasing popularity of video-sharing platforms as sources of health information, the quality and reliability of Marfan syndrome-related videos remain largely unknown (Liang et al., 2025; Qi et al., 2025; Zhu et al., 2026). As a rare hereditary connective tissue disorder with potentially life-threatening cardiovascular complications, delayed diagnosis or misinformation may have serious clinical consequences. Therefore, evaluating the quality of publicly available educational videos is particularly important for improving public awareness and facilitating timely medical consultation.

The primary objective of this study was to evaluate the educational quality, reliability, and content completeness of Marfan syndrome-related videos available on Bilibili and Douyin using validated assessment instruments. As a secondary, exploratory aim, we examined whether the publicly available interaction metrics of these videos are associated with their informational quality.

## Methods

### Study design and data collection

In this cross-sectional study, we identified Marfan syndrome-related videos by searching Bilibili (http://www.bilibili.com) and Douyin (the Chinese version of TikTok; http://www.douyin.com) on 25 April 2026. The keyword employed was the standard Chinese designation for Marfan syndrome, “马凡综合征”, which corresponds to the phrase Chinese viewers most commonly type when seeking this information. To minimize personalization bias, the searches were executed from freshly registered user accounts in anonymous browsing sessions, without any prior viewing history or logged-in profile. The default result ranking was applied without additional sorting or filtering. The results were then browsed continuously until no further videos appeared. All videos returned in were subsequently screened against the predefined inclusion and exclusion criteria described below. Because social media platforms continuously refresh their content libraries and ranking algorithms, screening and data collection were completed on the same day to minimize temporal variation in video availability (Huang et al., 2024).

### Inclusion and exclusion criteria

To be included, a video had to concentrate primarily on Marfan syndrome. The exclusion criteria comprised (1) videos that duplicated another item already captured; (2) videos judged irrelevant to Marfan syndrome or intended mainly to promote specific products or clinics; (3) videos shorter than 10 s; and (4) videos uploaded within the week preceding the day of data collection. When duplicates were detected, the version uploaded earliest was retained, and the videos that remained after screening constituted the analytic sample for each platform.

### Data extraction

For every video that passed screening, the following items were entered into a standardized Microsoft Excel spreadsheet: the platform (Bilibili or Douyin), the unique video URL, the upload date, the duration in seconds, and the numbers of likes, comments, shares, and collections displayed on the video page. All of these data were publicly accessible online. The uploaders of a video were regarded as the producers of its content, and each uploader was assigned to one of three categories. Accounts held by healthcare professionals, such as cardiologists, were classified as “Professionals” when the professional titles stated on the account, platform-verified professional badges (if available), hospital affiliations, or other public indications of medical practice supported this designation. Official accounts operated by hospitals, medical schools, healthcare organizations, or academic societies were classified as “Institutions”. Accounts belonging to lay users, patients, or their family members were classified as “Individuals”. The detailed classification guide is provided in Table S1. Ambiguous cases were reviewed independently by both reviewers and resolved through discussion or, if necessary, by a final decision of the third reviewer. In addition, the time since publication (the interval in days between the upload date and the date of data collection) was derived for each video, so that the length of time each video had been available to accumulate interactions could be taken into account.

### Video quality assessment

All eligible videos were then rated independently by the two authors, who served as the reviewers, using the Global Quality Scale (GQS), the modified Decision-making Information Support Criteria for Evaluating the Reliability of Non-randomised Studies (mDISCERN), the Journal of the American Medical Association (JAMA) benchmark criteria, and the Content Completeness Score (CCS). Both reviewers have sufficient experience in evidence-based medicine, critical appraisal of the medical literature, and the evaluation of health information. The ratings given by the two reviewers were compared for every video, and disagreements were discussed between them. When consensus could not be reached, an independent senior anesthesiologist and critical care physician experienced in perioperative cardiovascular management was consulted to make the final decision. The agreement between the two reviewers was quantified with Cohen’s quadratic weighted kappa (κ), with values below 0.00 interpreted as poor, 0.00–0.20 as slight, 0.21–0.40 as fair, 0.41–0.60 as moderate, 0.61–0.80 as substantial, and 0.81–1.00 as almost perfect (Table S2) (Cohen, 1968; Landis & Koch, 1977).

### Global quality scale

Overall informational quality was rated with the GQS, a five-point scale on which 1 denotes poor quality and 5 denotes excellent quality (Table S3) (Bernard et al., 2007).

### mDISCERN

To appraise the reliability of the health information, we used mDISCERN, an instrument adapted from the original DISCERN tool for consumer health information; it examines five items, namely whether the aims of the content are clear, whether reliable sources are used, whether the information is balanced, whether further sources of information or support are offered, and whether areas of uncertainty are acknowledged, with each item scored 0 or 1 to give a possible total of 0 to 5 (Table S4) (Charnock et al., 1999; Singh, Singh & Singh, 2012).

### The Journal of the American Medical Association benchmark criteria

Transparency and accountability were assessed with the JAMA benchmark criteria, which cover the four domains of authorship, attribution, disclosure, and currency, each scored 0 or 1 for a total between 0 and 4 (Table S5) (Silberg, Lundberg & Musacchio, 1997).

### Content completeness score

Disease-specific content completeness was measured with the CCS, which rates how fully six domains are explained, namely epidemiology, etiology, symptoms, diagnosis, treatment, and prognosis, assigning 0 (not explained), 1 (partly explained), or 2 (fully explained) to each domain and thus yielding a total between 0 and 12 (Nason, Donnelly & Duncan, 2016). Because these four validated instruments gauge different dimensions of the videos, using them together was intended to provide a comprehensive assessment of content quality.

### Ethical considerations

This study did not involve human participants or animals. All of the material analyzed consisted of publicly available videos on Bilibili and Douyin, and the investigators did not interact with any platform user; formal ethical approval was therefore not required. The large language model DeepSeek was used only to edit the language of this manuscript, and the manuscript contains no AI-generated content.

### Statistical analysis

Descriptive statistics were computed for the video characteristics and for the scores of the four assessment instruments. Because normality checks (Shapiro–Wilk tests, all *P* < 0.05, together with visual inspection of Q-Q plots) indicated that the continuous variables were not normally distributed, the results are reported as medians with interquartile ranges (IQR). The Mann–Whitney U test was used to compare continuous variables between the two platforms, and the Kruskal-Wallis test was used to assess differences across the three uploader categories. Effect sizes accompanied all non-parametric comparisons: for the Mann–Whitney U test, *r* = |*Z*|/√N, where *Z* is the standardized test statistic and N the total sample size; for the Kruskal–Wallis test, epsilon-squared was calculated as *ɛ*^2^ = (H−k + 1)/(N−k), where H is the Kruskal-Wallis statistic, k is the number of groups, and N is the total sample size. Spearman’s rank correlation was chosen for the association analyses due to non-normal nature of the data. Correlations were computed among video duration, interaction counts, and the quality scores, both for the pooled sample and for each platform separately. A two-sided *P* value below 0.05 was considered statistically significant, and all computations were performed in R software (version 4.3.2; R Core Team, 2023).

## Results

### Video selection process

Following the search strategy described in the Methods, 110 Douyin videos and 118 Bilibili videos judged relevant to Marfan syndrome were retrieved in total. After the exclusion criteria had been applied, 185 videos remained for analysis, of which 103 came from Douyin and 82 from Bilibili. The complete search and selection process is presented in Fig. 1.

**Figure 1:**
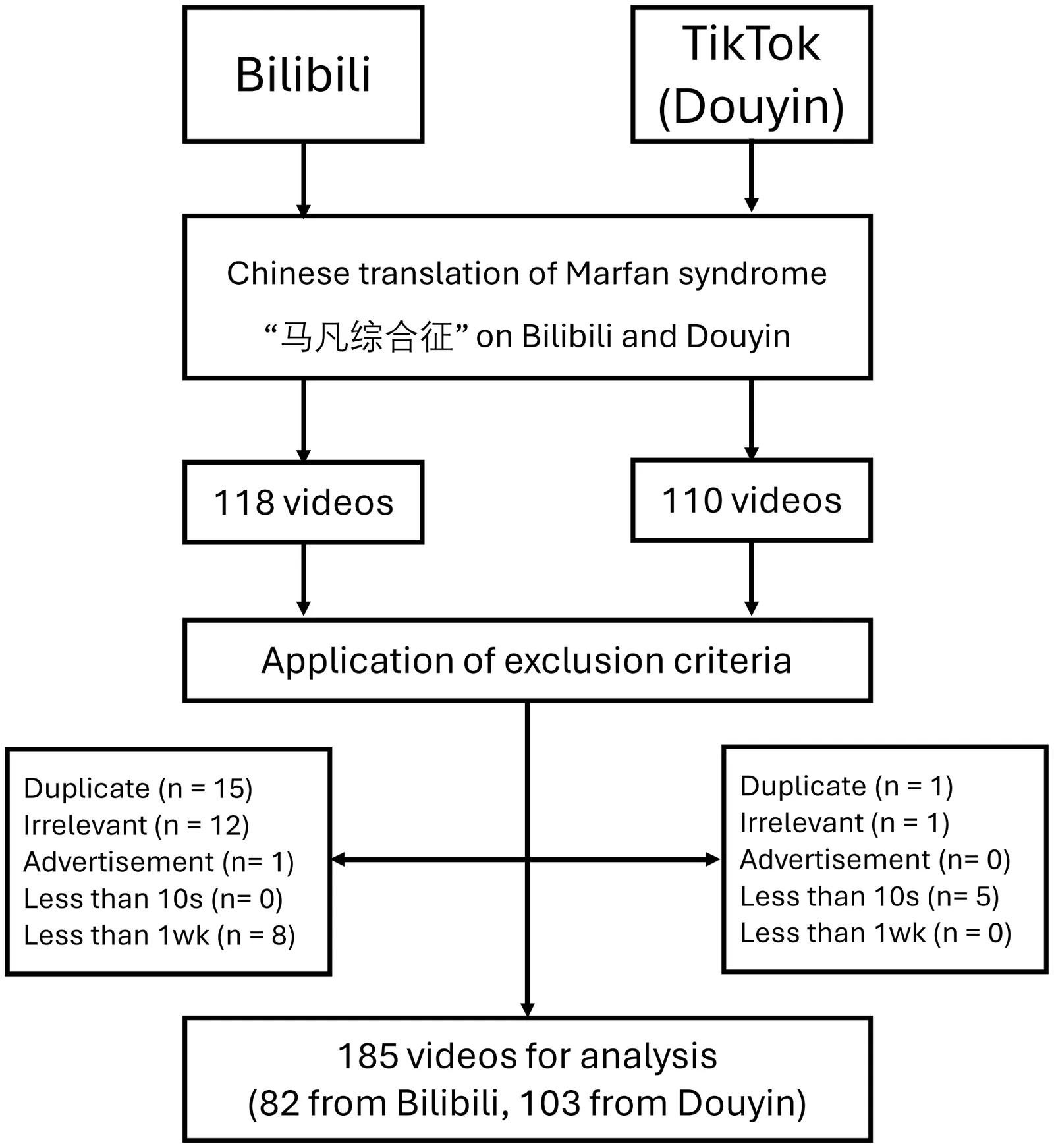
Search strategy and video selection process.

### Video characteristics

The basic characteristics of the included videos are summarized in Table 1. Video duration differed significantly between the two platforms: Bilibili videos were typically longer (median: 126.00 s (IQR: 59.00–377.00) *vs.* 64.00 s (IQR: 45.50–103.50); *P* < 0.001, *r* = 0.330). In contrast, Douyin videos displayed markedly higher absolute interaction counts: the median number of likes was 2,214.00 *vs.* 48.00 (*P* < 0.001, *r* = 0.393), comments 240.00 *vs.* 0.00 (*P* < 0.001, *r* = 0.522), shares 606.00 *vs.* 5.50 (*P* < 0.001, *r* = 0.559), and collections 475.00 *vs.* 24.00 (*P* < 0.001, *r* = 0.364). Because likes, comments, shares, and collections accumulate over the time a video has been online, the interval between publication and data collection was calculated so that the two platforms could be compared on a common basis. The results revealed that the time since publication also differed markedly between the two platforms: Bilibili videos had been available for a median of 747.50 days (IQR: 269.50–1,268.00), considerably longer than Douyin videos (median: 275.00 days (IQR: 128.00–642.00); *P* < 0.001, *r* = 0.266). Moreover, to account for this unequal exposure time, each interaction count was divided by the number of days since publication to derive per-day interaction rates, which were then compared between the platforms (Table 1). Even after this adjustment, Douyin videos continued to show significantly higher per-day interaction counts, with median likes per day of 8.73 *vs.* 0.15 (*P* < 0.001, *r* = 0.465), comments per day 1.04 *vs.* 0.00 (*P* < 0.001, *r* = 0.614), shares per day 2.21 *vs.* 0.01 (*P* < 0.001, *r* = 0.651), and collections per day 1.71 *vs.* 0.04 (*P* < 0.001, *r* = 0.440).

**Table 1:** Basic information of included videos from Bilibili and Douyin.

**Variables**	**Bilibili** **(*n* = 82)**	**Douyin** **(*n* = 103)**	**Statistic**	**Effect size (r)**	***P*** **-value**
Duration, seconds, M (Q_1_, Q_3_)	126.00 (59.00, 377.00)	64.00 (45.50, 103.50)	*Z* = −4.49	0.330	<0.001
Publication time, days, M (Q_1_, Q_3_)	747.50 (269.50, 1,268.00)	275.00 (128.00, 642.00)	*Z* = −3.62	0.266	<0.001
Likes, M (Q_1_, Q_3_)	48.00 (6.25, 3,509.25)	2,214.00 (491.50, 8,572.50)	*Z* = −5.34	0.393	<0.001
Likes/day, M (Q_1_, Q_3_)	0.15 (0.01, 5.17)	8.73 (1.70, 43.98)	*Z* = −6.33	0.465	<0.001
Collections, M (Q_1_, Q_3_)	24.00 (4.00, 552.25)	475.00 (160.50, 1,209.00)	*Z* = −4.95	0.364	<0.001
Collections/day, M (Q_1_, Q_3_)	0.04 (0.01, 1.71)	1.71 (0.50, 5.54)	*Z* = −5.98	0.440	<0.001
Comments, M (Q_1_, Q_3_)	0.00 (0.00, 79.75)	240.00 (48.00, 1,055.00)	*Z* = −7.10	0.522	<0.001
Comments/day, M (Q_1_, Q_3_)	0.00 (0.00, 0.15)	1.04 (0.19, 3.60)	*Z* = −8.35	0.614	<0.001
Shares, M (Q_1_, Q_3_)	5.50 (0.00, 87.25)	606.00 (98.00, 2,027.50)	*Z* = −7.60	0.559	<0.001
Shares/day, M (Q_1_, Q_3_)	0.01 (0.00, 0.18)	2.21 (0.48, 7.72)	*Z* = −8.85	0.651	<0.001
GQS, M (Q_1_, Q_3_)	3.00 (3.00, 3.00)	4.00 (3.00, 4.00)	*Z* = −5.33	0.392	<0.001
mDISCERN, M (Q_1_, Q_3_)	3.00 (2.00, 3.00)	2.00 (2.00, 3.00)	*Z* = −3.30	0.243	<0.001
JAMA, M (Q_1_, Q_3_)	2.00 (2.00, 3.00)	2.00 (2.00, 2.00)	*Z* = −1.16	0.085	0.245
CCS, M (Q_1_, Q_3_)	8.00 (7.00, 9.00)	8.00 (7.00, 10.00)	*Z* = −1.09	0.008	0.274

**Notes.**

M, median; Q_1_, 1st quartile; Q_3_, 3rd quartile; Z, Mann–Whitney test; GQS, Global Quality Scale; mDISCERN, modified Decision-making Information Support Criteria for Evaluating the Reliability of Non-randomised Studies; JAMA, Journal of the American Medical Association; CCS, Content Completeness Score.

**Figure 2:**
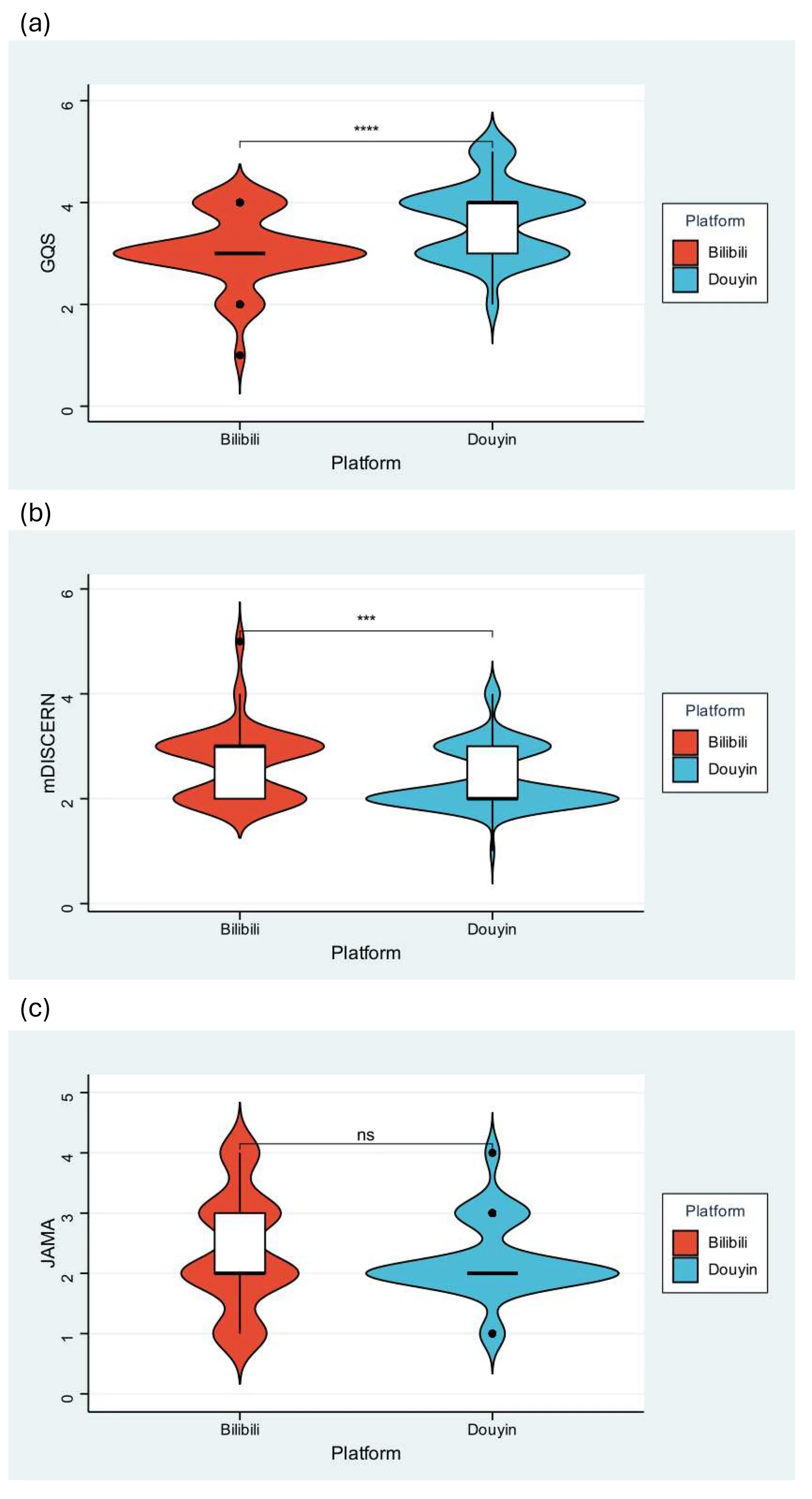
Violin plots for quality and distribution of Marfan syndrome-related videos across both platforms: (A) GQS, (B) mDISCERN, (C) JAMA. ns, non-significant (*P* ≥ 0.05); ***P < 0.001 and ****P < 0.0001.

### Video content, quality and reliability

Regarding video quality, Douyin videos achieved significantly higher GQS scores than Bilibili videos (median: 4.00 *vs.* 3.00; *P* < 0.001, *r* = 0.392), whereas the mDISCERN score was significantly higher on Bilibili (median: 3.00 *vs.* 2.00; *P* < 0.001, *r* = 0.243). No statistically significant differences were found for the JAMA benchmark criteria (median: 2.00 on both platforms; *P* = 0.245, *r* = 0.085) or the CCS (median: 8.00 on both platforms; *P* = 0.274, *r* = 0.080; Table 1). Agreement between the two reviewers, expressed as Cohen’s weighted kappa, was substantial to almost perfect for all four instruments: GQS κ = 0.621 (95% CI [0.512–0.713]), mDISCERN κ = 0.663 (95% CI [0.530–0.763]), JAMA benchmark criteria κ = 0.765 (95% CI [0.674–0.836]), and CCS κ = 0.842 (95% CI [0.802–0.874]; all *P* < 0.001; Table S2).

The violin plots in Fig. 2 further illustrate the distribution of the quality metrics on the two platforms. GQS scores were significantly higher on Douyin and clustered densely around four, whereas Bilibili scores were concentrated mainly at three. For mDISCERN, Bilibili outperformed Douyin, although the shapes of the two distributions were similar. The JAMA benchmark criteria overlapped considerably between the platforms, in keeping with the absence of a significant difference. Taken together, Douyin content tended to score higher on overall quality, Bilibili content appeared somewhat more reliable, and transparency was broadly comparable across the two platforms.

### Video sources and types

The composition of uploader sources differed markedly between the two platforms (*χ*2 = 49.28, *P* < 0.001; Fig. 3). Bilibili was predominantly supplied by institutional uploaders (53.66%, *n* = 44), followed by professionals (30.49%, *n* = 25) and individual users (15.85%, *n* = 13). On Douyin, by contrast, healthcare professionals were the dominant source (78.64%, *n* = 81), followed by individual users (11.65%, *n* = 12) and institutions (9.71%, *n* = 10).

**Figure 3:**
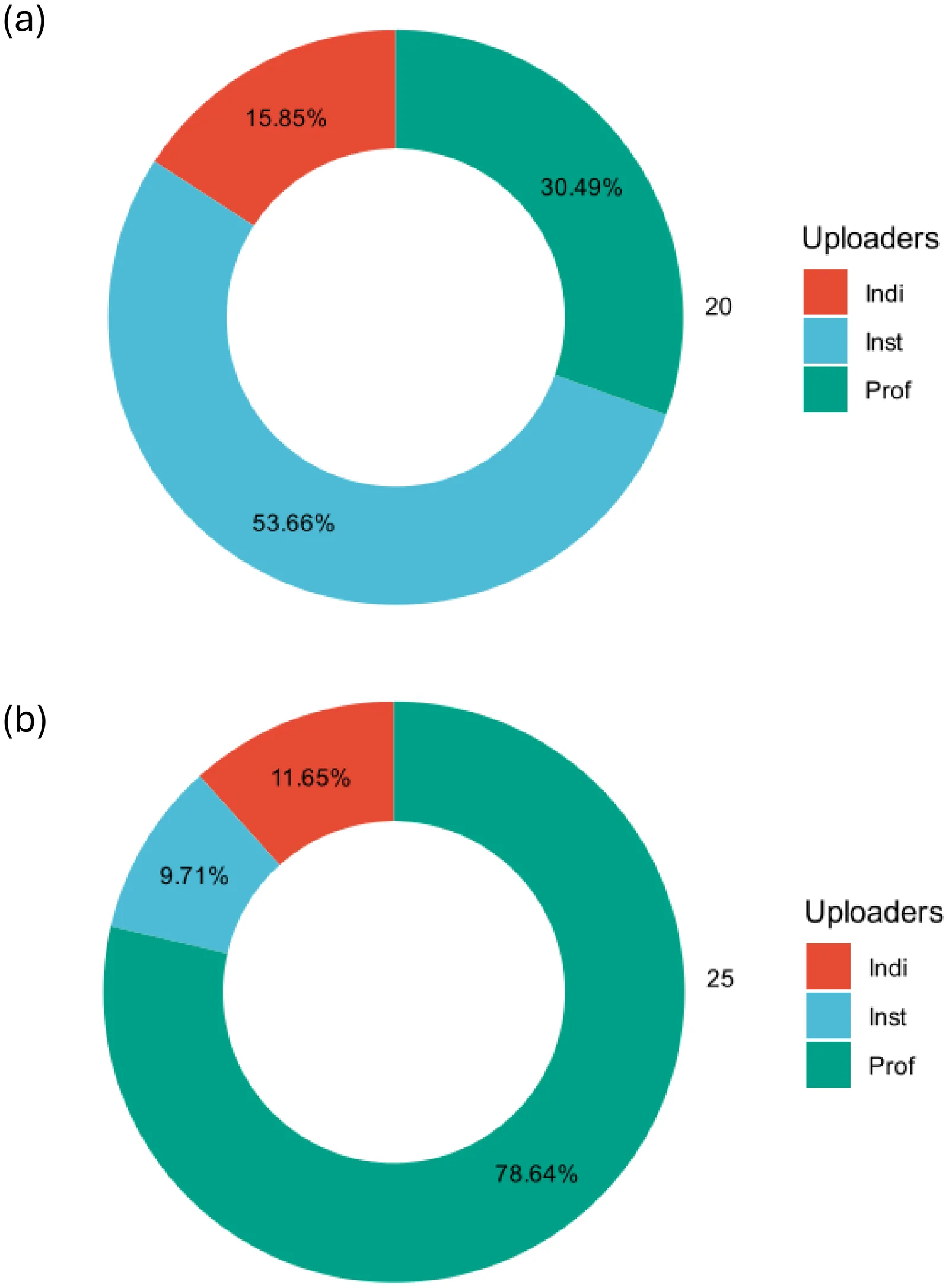
Distribution of Marfan syndrome-related videos by uploader type across both platforms. (A) Bilibili and (B) Douyin. Indi, Individuals, Inst, Institutions, Prof, Professionals.

Across the whole sample, professionals were the largest contributor group (*n* = 106), followed by institutional uploaders (*n* = 54) and individuals (*n* = 25; Table 2). Median video duration differed by uploader type: professionals produced the shortest videos (62.5 s) and institutions the longest (135 s; *P* < 0.001). The observed interaction counts, namely likes, collections, comments, and shares, did not differ significantly among the three groups. Quality scores, in contrast, varied significantly: professionals achieved the highest median GQS (4.00) and CCS (8.00), whereas individuals scored lowest on both instruments. mDISCERN scores also differed across uploaders (*χ*2 = 8.90, *P* < 0.05, *ɛ*2 = 0.038), but the JAMA benchmark criteria were comparable among the three groups.

Furthermore, the violin and ridge plots present the overall video quality across the three uploader categories (Fig. 4). The GQS scores of professionals clustered tightly between three and four, with a visible right tail extending to five, a pattern rarely observed among individual uploaders. Institutional content was concentrated mainly at three to four but spread less toward the top scores, whereas individual uploaders showed wider dispersion, with notable density at lower scores (2.00–3.00). For mDISCERN, professionals and institutions overlapped substantially around two and three, although professionals displayed a somewhat broader upper tail; individuals clustered near two and rarely reached high scores (Fig. 4B/4E). For the JAMA benchmark criteria, all three groups overlapped considerably around two and three, with professionals and institutions extending modestly toward higher values (3.00–4.00) and individuals again spreading toward lower scores (Figs. 4C/4F).

**Table 2:** Characteristics, content, quality, and reliability of Marfan syndrome-related videos by different uploaders on both social media platforms.

**Variables**	**Individuals** **(*n* = 25)**	**Institutions** **(*n* = 54)**	**Professionals** **(*n* = 106)**	**Statistic**	***P*** **-value**	**Effect size (*ɛ*** ^**2**^ **)**
Duration, M (Q_1_, Q_3_)	107.00 (54.00, 355.00)	135.00 (67.75, 350.25)	62.50 (43.00, 102.00)	*χ*^2^= 24.46^#^	<0.001	0.124
Likes, M (Q_1_, Q_3_)	4692.00 (43.00, 42,000.00)	1244.00 (31.75, 7,467.00)	676.00 (62.50, 4,199.75)	*χ*^2^= 1.93^#^	0.382	0.000
Comments, M (Q_1_, Q_3_)	234.00 (0.00, 873.00)	35.50 (0.00, 230.75)	74.50 (2.75, 462.00)	*χ*^2^= 2.71^#^	0.258	0.004
Collections, M (Q_1_, Q_3_)	475.00 (40.00, 1,933.00)	205.50 (16.25, 1,174.75)	296.50 (14.00, 851.75)	*χ*^2^= 0.68^#^	0.713	0.000
Shares, M (Q_1_, Q_3_)	212.00 (9.00, 1,897.00)	51.50 (5.50, 504.25)	240.00 (5.25, 1,421.00)	*χ*^2^= 3.97^#^	0.138	0.011
GQS, M (Q_1_, Q_3_)	3.00 (2.00, 3.00)	3.00 (3.00, 3.00)	4.00 (3.00, 4.00)	*χ*^2^= 40.14^#^	<0.001	0.209
mDISCERN, M (Q_1_, Q_3_)	2.00 (2.00, 2.00)	3.00 (2.00, 3.00)	2.00 (2.00, 3.00)	*χ*^2^= 8.90^#^	0.012	0.038
JAMA, M (Q_1_, Q_3_)	2.00 (2.00, 2.00)	2.00 (2.00, 3.00)	2.00 (2.00, 3.00)	*χ*^2^= 5.39^#^	0.068	0.019
CCS, M (Q_1_, Q_3_)	7.00 (5.00, 8.00)	8.00 (7.00, 9.00)	8.00 (7.00, 10.00)	*χ*^2^= 11.68^#^	0.003	0.053

**Notes.**

M, median; Q_1_, 1st quartile; Q_3_, 3rd quartile; #, Kruskal–Wallis test; GQS, Global Quality Scale; mDISCERN, modified Decision-making Information Support Criteria for Evaluating the Reliability of Non-randomised Studies, JAMA, Journal of the American Medical Association, CCS: Content Completeness Score.

**Figure 4:**
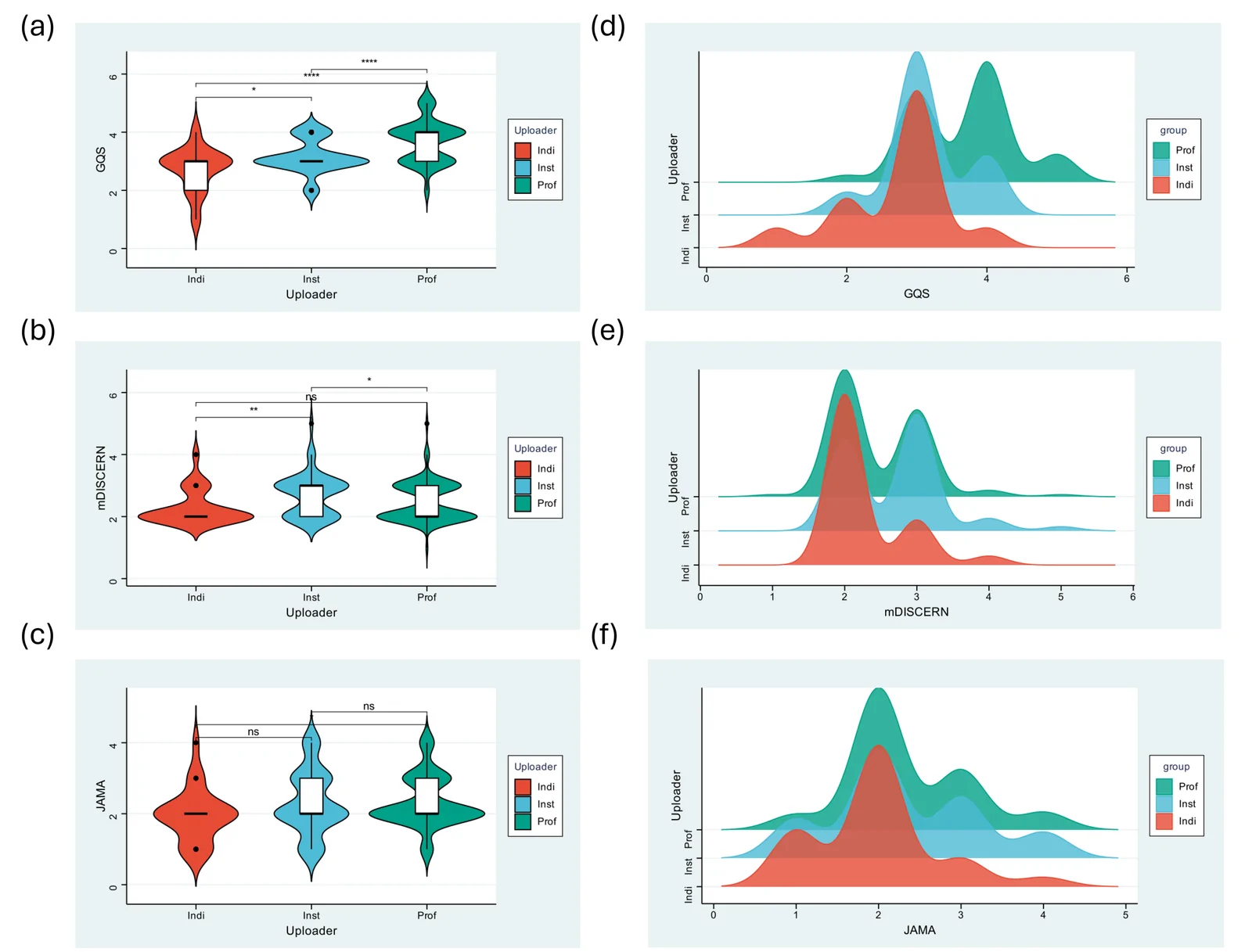
The violin and ridge plots of quality metrics distribution of videos related to Marfan syndrome across sources. (A/D) GQS, (B/E) mDISCERN, (C/F) JAMA. Indi, Individuals, Inst, Institutions, Prof, Professionals. ns: non-significant (*P* ≥ 0.05); *P < 0.05; **P < 0.01; and ****P < 0.0001.

### Correlation of interaction counts with video quality across platforms and uploader sources

Spearman correlation analyses were used to examine the relationships among video duration, interaction counts, and quality scores, and the results are displayed separately for each platform in Fig. 5. On Bilibili, the interaction counts were strongly intercorrelated (*r* = 0.85–0.92), pointing to synchronized patterns of user interaction (Fig. 5A). Their correlations with the quality measures were weak or inconsistent: GQS and mDISCERN correlated weakly and negatively with most interaction counts (*r* = −0.14 to −0.01), whereas JAMA scores showed mixed weak correlations (*r* = −0.18 to −0.08). CCS was moderately correlated with the other quality scores (*r* = 0.48–0.50) but only weakly with the interaction counts (*r* = −0.08 to 0.02).

**Figure 5:**
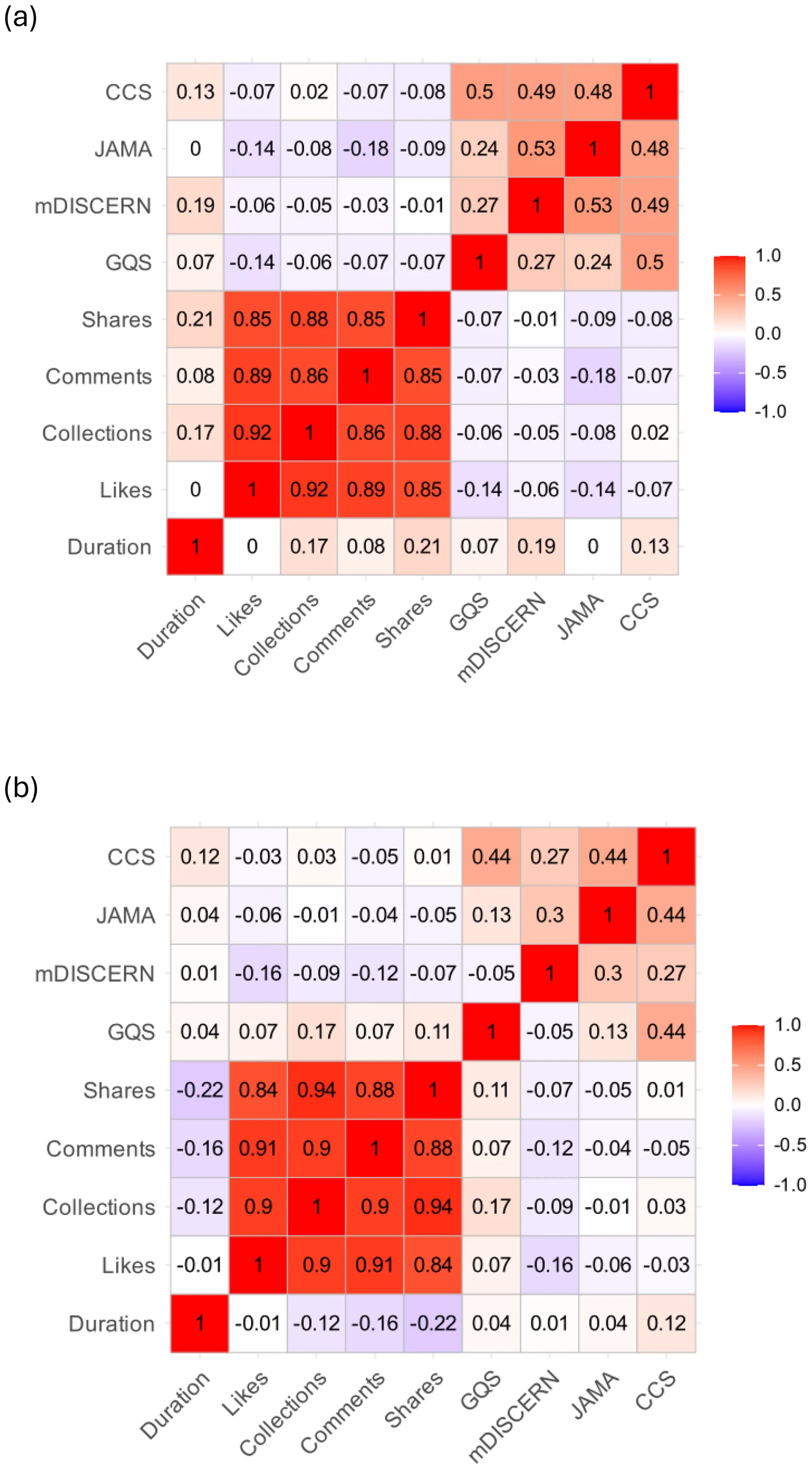
Correlation matrix of video engagement metrics and quality scores on both platforms. (A) Bilibili and (B) Douyin.

On Douyin, the interaction counts were likewise strongly intercorrelated (*r* = 0.84–0.94), suggesting highly synchronized user interaction patterns (Fig. 5B). Correlations between these counts and the quality scores were generally weak: GQS correlated weakly and positively (*r* = 0.07–0.17), mDISCERN negatively (*r* = −0.16 to −0.07), and the JAMA benchmark criteria weakly and negatively (*r* = −0.06 to −0.01). CCS correlated positively with the other quality measures (*r* = 0.27–0.44) but showed mixed, near-zero associations with the interaction counts (*r* = −0.03 to 0.03). Unlike on Bilibili, video duration on Douyin showed mixed associations with the interaction counts (*r* = −0.22 to 0.12). These findings collectively reveal a concerning pattern across both platforms: absolute interaction counts do not reliably reflect the informational quality and reliability of Marfan syndrome-related content, indicating that high popularity should not be interpreted as evidence of high educational value.

## Discussion

This study evaluated the educational quality, reliability, and content completeness of Marfan syndrome-related short videos on two major Chinese video-sharing platforms. Although a number of videos conveyed useful educational messages, the quality of the information varied considerably across platforms and uploader categories. Within this sample, observed interaction counts were only weakly associated with the quality measures, indicating that popularity should not be regarded as a reliable indicator of educational value.

The overall moderate GQS and mDISCERN scores indicate that most videos offered a basic level of educational value but did not deliver comprehensive, evidence-based information. This aligns with prior evaluations of rare disease-related social media content, such as videos about sarcoidosis and Takotsubo syndrome, which likewise reported suboptimal quality and reliability despite ready accessibility (Buschulte et al., 2024; Liang et al., 2022). For a complex multisystem genetic condition such as Marfan syndrome, such deficits are especially concerning, because effective management demands a detailed understanding of cardiovascular risk (such as aortic aneurysm and dissection), genetic counseling, lifestyle modification, and lifelong surveillance (Muino-Mosquera et al., 2020). Short-form videos inherently prioritize brevity and visual appeal over depth, so oversimplified or incomplete messages are a genuine risk. Consequently, such content is unlikely by itself to support clinical decision-making or patient self-management (Liu et al., 2026; Peng et al., 2025).

The findings also underscore the decisive role of content creators in determining information quality. Videos uploaded by healthcare professionals received significantly higher GQS and mDISCERN scores than those from non-professional sources, consistent with earlier evidence that a professional background strongly predicts content accuracy and reliability (Cao et al., 2025; Zhao & Liu, 2026). Institutional accounts achieved the highest scores on the JAMA benchmark criteria, which may reflect closer adherence to transparency and accountability standards among institutional uploaders. However, the highest-quality videos were not always the most prominent or widely viewed, again indicating a disconnect between informational value and audience reach.

Clear platform-specific patterns also emerged. Douyin videos were shorter but accumulated far higher absolute interaction counts, whereas Bilibili videos were longer and achieved slightly better transparency. These contrasts may reflect the distinct design and user behavior patterns of the two platforms. Cross-platform comparisons of these absolute counts should be interpreted cautiously: Douyin and Bilibili differ markedly in user population size, recommendation algorithms, and interaction mechanisms, so the absolute counts mirror platform-specific ecosystems rather than intrinsic differences in content quality. Douyin’s algorithm prioritizes rapid consumption and virality, favoring concise and visually engaging clips, whereas Bilibili accommodates longer videos that allow more detailed, structured explanations. Platform-tailored communication strategies may therefore be warranted: Douyin appears well suited to awareness campaigns and the dissemination of key messages, while Bilibili may better serve in-depth patient education (Cai et al., 2024; Cakici & Koseoglu, 2025; Neugebauer et al., 2025).

A further observation is the near-absence of correlation between the interaction counts and the quality measures. Although the counts themselves were strongly intercorrelated, none was consistently associated with the GQS, mDISCERN, or JAMA scores, indicating that, for the Marfan syndrome-related content examined here, popularity did not reliably reflect informational quality. Comparable findings have been reported for other health topics and suggest that recommendation systems may amplify content on the basis of entertainment value or emotional resonance rather than informational quality (Liu et al., 2026; Peng et al., 2025; Zhao & Liu, 2026). This points to the value of stronger content moderation, greater algorithm transparency, and, potentially, the integration of quality signals into platform recommendation systems. We also noted the limited presence of patient-generated content in this study. Although personal narratives can offer valuable experiential insight and enhance relatability, their scarcity may reflect barriers to content creation or reduced visibility under platform algorithms; when such content does appear, it should be interpreted cautiously, as it may lack medical accuracy or generalizability (Cai et al., 2024).

The findings of this study also have broader implications for healthcare professionals and policymakers. Given the widespread use of social media for seeking health information, there is a clear need for increased professional engagement in these platforms (Xu et al., 2025). Healthcare providers should be encouraged to actively contribute high-quality, evidence-based content that is both accurate and accessible. Additionally, professional societies and institutions could play a leading role by developing standardized guidelines for online health communication, ensuring consistency and reliability (Zhe & Zheng, 2026). Educational interventions aimed at improving digital health literacy among the public are also essential, enabling users to critically evaluate the information they encounter (Zhe & Zheng, 2026). However, video-sharing platforms are primarily designed to provide concise and accessible public health information rather than comprehensive and professional clinical guidance. Therefore, our evaluation should be interpreted within the context of public health communication rather than as an assessment of their suitability for supporting independent clinical decision-making. High-quality short videos may effectively improve disease awareness and encourage timely medical consultation, although they cannot replace professional healthcare advice (Huang et al., 2024).

This study has several strengths. Firstly, it addresses a critical gap by investigating the quality of information for Marfan syndrome, a rare but life-threatening multisystem disorder where accurate patient education is vital for informed decision-making and adherence to lifelong multidisciplinary management. Secondly, four validated instruments (the GQS, mDISCERN, the JAMA benchmark criteria, and the CCS) were applied within a comparative dual-platform design, allowing the quality and reliability of videos on two widely used Chinese video-sharing platforms to be assessed systematically. Thirdly, the study examined, within this sample, whether observed interaction counts were associated with the quality measures; these findings may help inform future efforts to improve search algorithms and content moderation so that reliable medical content is promoted.

However, several limitations should be acknowledged. First, the cross-sectional design with single day data collection offered only a snapshot of a rapidly changing environment; longitudinal data collection at multiple time points would better capture temporal shifts in video availability, content quality, and viewer interactions. Second, the use of a single Chinese search term and the restriction to Chinese-language videos on Douyin and Bilibili may have limited the completeness of video retrieval and the generalizability of the findings to other search terms, languages, platforms, and cultural settings; future studies that incorporate multiple search keywords, additional platforms, and multilingual samples would improve representativeness. Third, only publicly available interaction counts were analyzed, whereas other proprietary indicators that could serve as denominators, such as view counts, watch time, completion rates, and audience retention, were inconsistently available; normalized interaction metrics (for example, per view or per minute of watch time) would permit more direct cross-platform benchmarking. Although we compared the time since publication between the two platforms, interaction counts would ideally be modified as per unit of exposure time. Fourth, the classification of uploaders relied mainly on publicly accessible profile information and may thus have involved some degree of misclassification; this risk was reduced by independent assessment of ambiguous accounts by both reviewers, with any remaining discrepancies resolved through discussion or adjudication by the third reviewer. Fifth, the quality ratings were based on the judgment of only two primary reviewers, with the third reviewer involved solely as an adjudicator for unresolved cases. Although the weighted kappa coefficients indicated substantial agreement, such statistics measure consistency between raters and do not transform expert-based ratings into directly observed, objective variables; the limited number of raters and the inherent subjectivity of these instruments constrain the objectivity and generalizability of the quality ratings, and additional independent raters would have strengthened the design. These instruments also capture educational quality, reliability, transparency, and completeness rather than the clinical accuracy of the medical content, which was not verified by Marfan syndrome specialists. No amount of statistical testing can compensate for these limitations of the underlying observational data. Finally, we assessed only the characteristics of the videos themselves and could not evaluate viewers’ comprehension, information retention, behavioral changes, health-care-seeking behavior, or subsequent clinical outcomes resulting from exposure to the videos.

## Conclusions

In conclusion, Marfan syndrome-related videos on Douyin and Bilibili showed variable educational quality and reliability. Videos uploaded by healthcare professionals generally earned higher quality scores; however, within the videos analyzed here, observed interaction counts were weakly or negligibly associated with the quality measures, so popularity should not be interpreted as evidence of high informational quality. These findings reinforce the importance of encouraging evidence-based health communication and of strengthening digital health literacy among both content creators and audiences. Future studies involving additional platforms and longitudinal designs are warranted.

## Supplemental Information

10.7717/peerj.21843/supp-1 Supplemental Information 1 Supplemental Tables 1-5

10.7717/peerj.21843/supp-2 Supplemental Information 2 Raw data for included videos Platforms, Uploaders are categorical data. Duration, Likes, Collections, Comments, and Shares are numerical data. GQS, mDISCERN, JAMA, CCS are ordinal data.

10.7717/peerj.21843/supp-3 Supplemental Information 3 STROBE Checklist

## Additional Information and Declarations

### Data Availability

The following information was supplied regarding data availability: The raw measurements are available in the Supplemental Files.
