# Supplementary material for: The quality and reliability of short videos about Marfan syndrome on Bilibili and Douyin: a cross-sectional study
Source: PeerJ. 2026 Oct 25;14:e21843. doi: 10.7717/peerj.21843 (PMC13715633; doi:10.7717/peerj.21843)
Supplement: Supplementary file 1 [file peerj-14-21843-s001.docx]

**Supplementary Materials**

**Supplementary Table 1.** Uploader Classification

| **Category** | **Definition** | **Key Criteria** | **Examples** |
| --- | --- | --- | --- |
| Professionals | Individual medical professionals (such as physicians and nurses) who create health-related content in a professional capacity. | Self-identification as physicians in channel/profile description  Verified professional credentials  Content reflects clinical expertise | Dr. Gang Li,  Nurse Wang,  Verified account on Bilibili/Douyin with official badge |
| Institutions | Organizations, agencies, or entities that produce or disseminate health information. | Channel affiliated with an organization  Official verification badge  Content produced by a team | World Health Organization (WHO)  Centers for Disease Control and Prevention (CDC)  XXX Hospital |
| Individuals | Non-professional users without institutional or medical affiliation.  Patients, family members, and lay users. | No professional or organizational affiliation disclosed  No medical/institutional verification  Content primarily personal perspective or anecdotal | My MS Journey  Mom shares son's MS symptoms  General user without credentials posting like "what you need to know about MS" |

**Coding Notes:** Ambiguous cases would be determined by the third reviewer if two reviewers’ discussion did not reach consensus.

**Supplementary Table 2.** The inter-rater reliability of video quality scores

|  | **GQS (95% CI)** | **mDISCERN (95% CI)** | **JAMA (95% CI)** | **CCS (95% CI)** |
| --- | --- | --- | --- | --- |
| **Cohen’s weighted kappa coefficients** | 0.621 (0.512, 0.713) | 0.663 (0.530, 0.763) | 0.765 (0.674, 0.836) | 0.842 (0.802, 0.874) |
| **Z-statistic** | 15.72 | 16.80 | 18.56 | 18.21 |
| ***P*-value** | < 0.001 | < 0.001 | < 0.001 | < 0.001 |

GQS: Global Quality Scale, CI: Confidence Interval, mDISCERN: Modified Decision-making Information Support Criteria for Evaluating the Reliability of Non-randomised Studies, JAMA: Journal of the American Medical Association, CCS: Content Completeness Score.

**Supplementary Table 3.** The GQS quality criteria.

| **Item features** | **Points** |
| --- | --- |
| Poor quality; poor flow of the videos; most information missing; not at all useful for patients | 1 |
| Generally poor quality; some information listed, but many important topics missing; of very limited use to patients | 2 |
| Moderate quality; suboptimal flow; some important adequately discussed, but other information poorly discussed; somewhat useful for patients | 3 |
| Good quality and generally good flow; most of the relevant information listed, but some topics not covered; useful for patients | 4 |
| Excellent quality and flow; very useful for patients | 5 |

GQS: Global Quality Score

**Supplementary Table 4.** The mDISCERN quality criteria.

| **Reliability Score** |  |
| --- | --- |
| 1. Is the video clear, concise, and understandable? | Yes (1) / No (0) |
| 2. Are valid sources cited? | Yes (1) / No (0) |
| 3. Is the content presented balanced and unbiased? | Yes (1) / No (0) |
| 4. Are additional sources of content listed for patient reference? | Yes (1) / No (0) |
| 5. Are areas of uncertainty mentioned? | Yes (1) / No (0) |

mDISCERN: Modified DISCERN

**Supplementary Table 5.** The JAMA benchmark criteria

| **Criteria**  **(1 point for each item)** | **Description** |
| --- | --- |
| **Authorship** | Author and contributor credentials and their affiliations should be provided |
| **Attribution** | Clearly lists all copyright information and states references and sources for content |
| **Currency** | Initial date of posted content and subsequent updates to content should be provided |
| **Disclosure** | Conflicts of interest, funding, sponsorship, advertising, support, and video ownership should be fully disclosed |

JAMA: Journal of the American Medical Association
